# Artificial Neural Network Algorithms for 3D Printing

**DOI:** 10.3390/ma14010163

**Published:** 2020-12-31

**Authors:** Muhammad Arif Mahmood, Anita Ioana Visan, Carmen Ristoscu, Ion N. Mihailescu

**Affiliations:** 1 Laser Department, National Institute for Laser, Plasma and Radiation Physics (INFLPR), 077125 Magurele, Ilfov, Romania; arif.mahmood@inflpr.ro (M.A.M.); anita.visan@inflpr.ro (A.I.V.); 2 Faculty of Physics, University of Bucharest, 077125 Magurele, Ilfov, Romania

**Keywords:** additive manufacturing, 3D printing, artificial neural networks, algorithms

## Abstract

Additive manufacturing with an emphasis on 3D printing has recently become popular due to its exceptional advantages over conventional manufacturing processes. However, 3D printing process parameters are challenging to optimize, as they influence the properties and usage time of printed parts. Therefore, it is a complex task to develop a correlation between process parameters and printed parts’ properties via traditional optimization methods. A machine-learning technique was recently validated to carry out intricate pattern identification and develop a deterministic relationship, eliminating the need to develop and solve physical models. In machine learning, artificial neural network (ANN) is the most widely utilized model, owing to its capability to solve large datasets and strong computational supremacy. This study compiles the advancement of ANN in several aspects of 3D printing. Challenges while applying ANN in 3D printing and their potential solutions are indicated. Finally, upcoming trends for the application of ANN in 3D printing are projected.

## 1. Introduction

Three-dimensional (3D) printing, under additive manufacturing, is a new, promising field that has gained prevalent attention in all fields [1,2,3,4]. It involves layer-by-layer deposition via a computer-aided design (CAD) model. 3D printing has several advantages: (a) it can produce parts with complex shapes, which are difficult to produce using conventional manufacturing processes; (b) it can manufacture parts with novel characteristics [5]; and (c) it decreases the material surplus, reducing the manufacturing cost. These are the reasons why 3D printing has become popular in a very short timeframe. However, 3D-printed parts also contain defects that are very different from those generated by conventional methods. There are various types of defects, including porosity, anisotropy in the microstructure, and part distortion, resulting from high residual stresses due to rapid heating and slow conduction [6]. Hence, it is essential to understand the correlation among the material metallurgical characteristics, printing parameters, microstructure, and properties of 3D-printed parts. There are various essential parameters, e.g., laser power, laser scanning speed, hatch distance between two adjacent layers, powder flow rate, etc., which influence the properties of the final produced parts. Inappropriately, the link between process parameters and the quality of the printed part is very complex and therefore difficult to understand.

3D printing is a complex multi-physics process. One of the ways to better understand this process is to simulate it before performing any experiment. Various studies have been carried out to identify the relation between process parameters and the printed workpiece. Acharya et al. [7] combined computational fluid dynamics and phase-field models to simulate grain formation for the powder bed fusion process based on the primary operating parameters, including laser power and scanning speed. Fergani et al. [8] presented a mathematical model to analyze the residual stress distribution within the 3D printing of metallic parts, while Chen et al. [9] developed a finite-element (FE) analysis model to examine melt-pool dimensions and the deposited layer profile. Wang and Li [10] developed an accurate simulation method for a fused deposition modeling (FDM)-printed monolayer shape memory polymer (SMP) based on the secondary development of the ABAQUS software, which could accurately and simply simulate the deformation processes of various preprogrammed structures. Based on the constitutive model, the degree and mode of model deformation due to external temperature were simulated by configuring the anisotropic pre-strain stored in the printed model. As a result, the deformation of the SMP models controlled by structural parameters was consistent with the simulation results. Self-folding origami structures controlled by structural factors were designed by the proposed simulation method. The origami structures folded according to the predicted deformation angle and direction under thermal stimulation. This method was able to accurately simulate the deformations of complex 3D models with different structural parameters. Wu et al. [11] developed, in the case of stereolithography, analytical, numerical, and experimental methods to characterize the curing conversion of a mask-exposed ceramic slurry. Visualization of the curing conversion profiles was achieved through optical microscopic observation of partially de-bound green parts. The results showed that the curing conversion in the green body was step-like and nonuniform between and within layers because of the attenuation of UV light. The most considerable difference in the curing conversion appeared at the layer bonding interface. Uniform curing conversion should be achieved to reduce the layered properties of printed ceramics. 

Zhang and Chou [12] developed a 3D FE model to simulate the FDM melt-pool process. The same model was used and improved by Zhang in [13] for the simulation of residual stresses to evaluate part distortion. Prototype parts were built and used to validate the simulated results. Bellini et al. [14] and Venkataraman et al. [15] analytically modeled the material flow on an extrusion nozzle. Jee and Sachs [16] proposed a visual simulation technique to facilitate surface texture designs to be produced by the material jetting process (MJP). This technique simulates the MJP by taking into consideration all the necessary geometric attributes of physical phenomena and therefore enables the achievement of a manufacturable design with minimum iterations. Sachs and Vezzetti [17] numerically modeled the deposition process of a new MJP head design to ensure a reliable and continuous jet deposition, resulting in an order of magnitude increase in the printing speed. Curodeau [18] modeled the drop-merging process (the phenomenon where, in a uniformly spaced train of drops, the leading drop is retarded by air drag and tends to merge with the drop behind it) to evaluate the number of merged drops for various distances and printing conditions. One can conclude from the studies mentioned above that the simulations usually vary from microscale to macroscale and focus on one or two features only, eliminating an in-depth understanding of the 3D printing process. It is unfeasible to forecast the 3D printing process effectively and in a very short period.

Various data-driven models, known as machine learning, have been adopted widely in the domain of 3D printing. The main advantage of such models is that there is no need to develop a long list of multi-physics equations. Instead, they automatically learn the correlation between the inputs and outputs based on the data provided for training. In machine learning, artificial neural network (ANN) is the most commonly used algorithm and is under continuous development due to the availability of enormous databases and computational resources [19]. For instance, ANNs are continuously evolving in the fields of machine vision [20], vocal recognition [21], language processing [22], and self-governing driving [23]. Moreover, there is a new trend for implementing ANN in the 3D printing field. This article provides an overview of the current progress of ANN implementation in the field of 3D printing. It has been classified in the following ways: Section 2 provides a comprehensive introduction of 3D printing technologies, Section 3 discusses the introduction of ANN, Section 4 compiles the applications of ANN in the field of 3D printing, Section 5 discusses the potential challenges and their solutions for ANN implementation, and Section 5 describes the future trends for the implementation of ANN in the 3D printing field.

## 2. Three-Dimensional (3D) Printing Processes

Additive manufacturing (AM) is the general term for technologies that, based on a geometrical representation, fabricate physical objects by the successive addition of material. Three-dimensional (3D) printing lies under the category of AM. These technologies are presently used for various applications in the engineering industry as well as other areas of society, such as medicine, education, architecture, cartography, toys, and entertainment. According to the standard ISO/ASTM 52900:2015 [24], the 3D printing process is classified into seven categories. Table 1 compiles the different 3D printing processes.

Each 3D printing process comprises the following steps [25,26].

- Firstly, CAD software is used to develop a CAD model.
- This CAD model is converted into stereolithography format (.STL), which is the wedge-shaped drawing of a 3D CAD model.
- Then, the file is sliced into several thin cross-sectional layers, using slicing software known as a slicer.
- Further, the part is printed by a 3D printer using computer numerical control (CNC) codes developed from the sliced file. CNC codes define the smooth and jerk-free movements of the deposition head, giving efficient and better-quality results [27,28].
- Finally, postprocessing steps, including surface treatments, sintering, or finishing, are usually performed [29].

### 2.1. Binder Jetting

**Figure 1:**
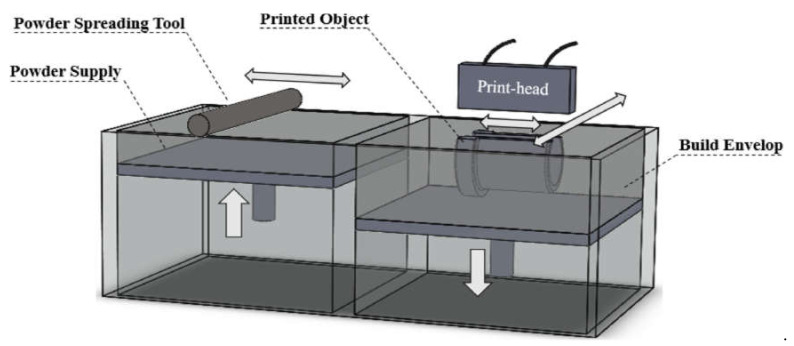
Schematic of the binder jetting system [30]; with permission from Elsevier.

Figure 1 illustrates a typical binder jet system [30]. For each layer of the part, a layer of powder is spread, typically using a counterrotating roller. Afterward, an inkjet print-head pour/flows the liquid binding agent to the powder bed to generate the 2D pattern for the layer. Some binder/powder systems may use heaters to help control moisture and curing, but heat is not an essential process requirement. After each layer, the build platform is lowered to make room for the next layer, and the process is repeated. The as-printed parts are fragile and typically require postprocessing to improve the mechanical properties [30].

### 2.2. Direct Energy Deposition

**Figure 2:**
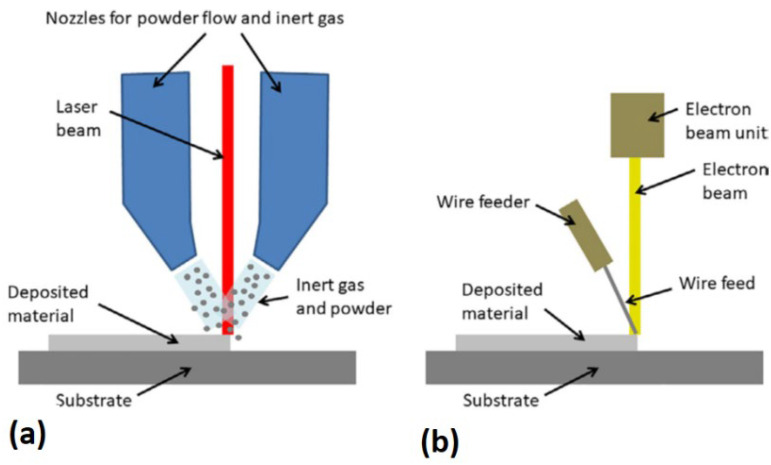
Schematics of two direct energy deposition (DED) systems: (**a**) laser with powder feedstock and (**b**) an electron beam with wire feedstock [31]; with permission from Elsevier.

Figure 2 shows a schematic of the direct energy deposition (DED) process [31]. The heat input can either be a laser, electron beam, or plasma arc. The material feedstock is either a metal powder or wire. Powders result in lower deposition efficiency compared with metal wires, as only part of the total powder is melted and bonded to the substrate. Powder DED machines often include an inert gas blown together with the powder from the nozzles, thereby sheathing the melted region, reducing the oxidization rate. Powder DED systems can use single or multiple nozzles to eject the metal powders. The use of multiple nozzles allows for the possibility of mixing different materials to get functionally graded materials [31].

### 2.3. Material Extrusion

**Figure 3:**
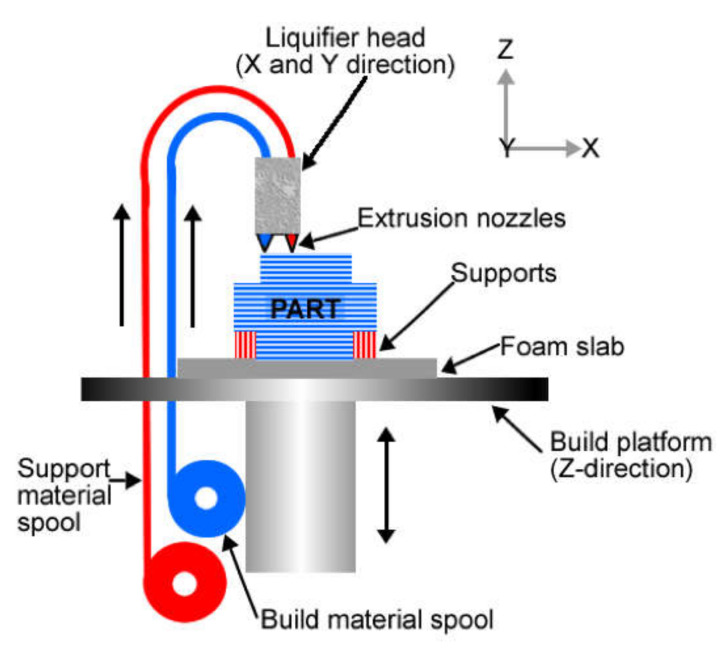
Schematic of material extrusion process [32]; published under the open access Creative Commons license with MDPI.

In material extrusion, the material is extruded, and a layer-by-layer part is built from a CAD file. A schematic of this process is shown in Figure 3 [32]. This technique allows flexibility in design, which is undoubtedly beneficial, e.g., for implant fabrication, because implant size and shape can be tailored, leading to the production of customized patient products [32]. 

### 2.4. Material Jetting

**Figure 4:**
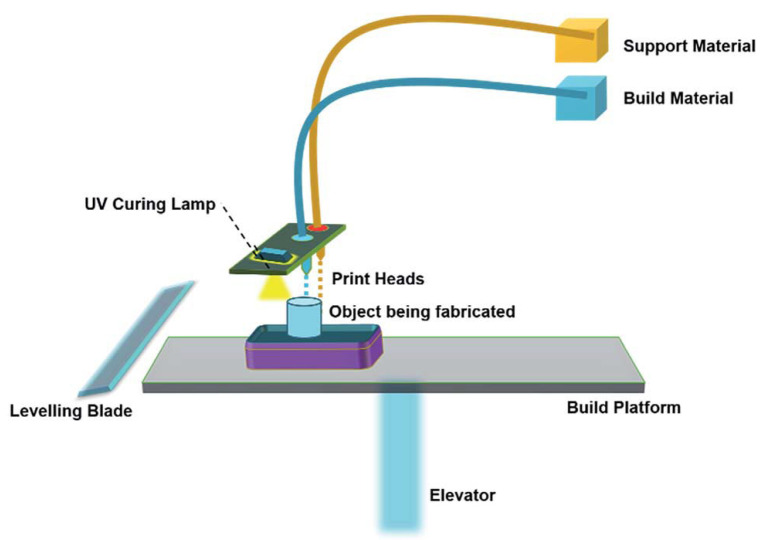
Schematics of the material jetting process [33]; published under the open access Creative Commons license with the Royal Society of Chemistry.

A schematic of material jetting is shown in Figure 4 [33]. Several investigations have been carried out on two modifications of inkjet printers, namely continuous inkjet printing (CIJ) and drop-on-demand (DOD). The unique difference between CIJ and DOD is the timing of droplet generation. In DOD, droplets are generated when required, whereas, in CIJ, the droplets are generated by breaking up the continuous stream of droplets through an ejection nozzle. In all AM technologies, material jetting is the only technology that offers the highest *z*-direction resolution with layer thicknesses as low as 16 mm. Materials such as acrylonitrile butadiene styrene (ABS), polyamide, polylactic acid (PLA), and their composites are commonly used for printing 3D objects by CIJ and DOD [33].

### 2.5. Powder Bed Fusion

**Figure 5:**
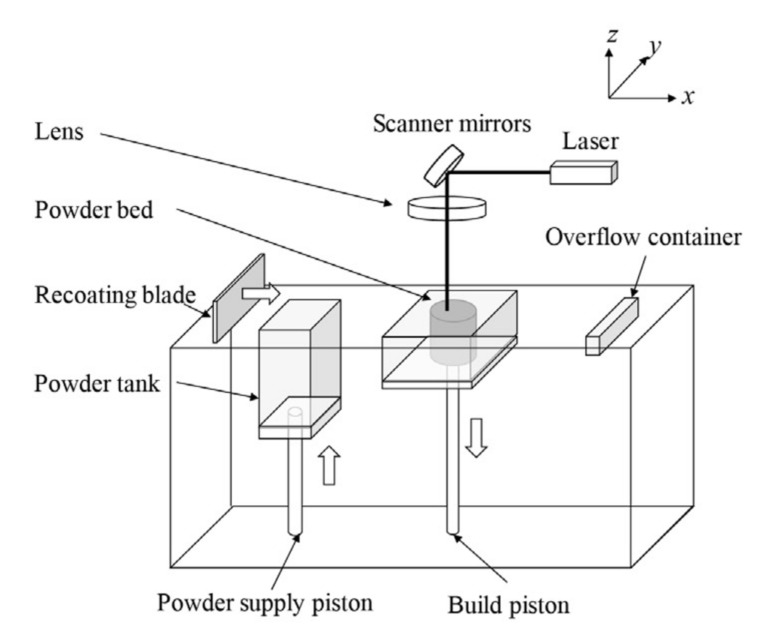
Schematic of powder bed fusion equipment [34]; with permission from Elsevier.

Powder bed fusion (PBF) uses a high-energy power source to melt or sinter a metallic powder bed selectively. A schematic of the PBF setup is shown in Figure 5 [34]. In this process, the laser beam passes through a system of lenses and is reflected by a mirror onto the platform surface. The mirrors are used to control the laser beam spot movement in the planar (*x* and *y*) directions on the designed paths. The platform moves downward after a layer of powder is selectively melted, after which a recoating blade or brush pushes another layer of fresh powder from the powder dispenser to the top of the previously built surface, and the laser scanning process is repeated. The building chamber of the PBF machine is filled with an inert gas, argon in most cases, to avoid oxidization of the metal as it melts and resolidifies [34].

### 2.6. Sheet Lamination

**Figure 6:**
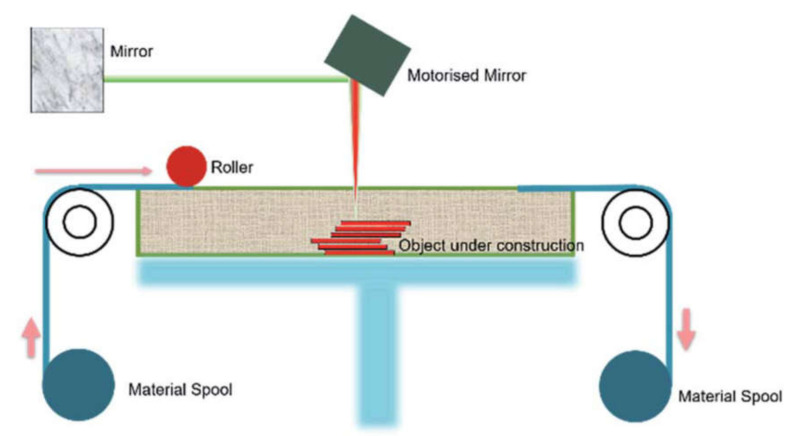
Schematic of the sheet lamination process [33]; published under the open access Creative Commons license with the Royal Society of Chemistry.

Sheet lamination, also known as laminated object modeling (LOM), manufactures objects and prototypes by cutting, sequentially laminating, and bonding. LOM works on a principle where thin adhesive-coated metallic sheets or layers of plastic are bonded together using ultrasonic welding and shaped by a laser cutter. A schematic of the sheet lamination process is shown in Figure 6 [33]. Since the process involves solid-state bonding and additional adhesives, the material is not required to reach its melting point for the bonding to occur. A variety of materials can be manufactured using sheet lamination, which includes paper, ceramics, metals (aluminum, stainless steel, copper, and titanium), plastics, fabrics, synthetic materials, and composites [33].

### 2.7. Vat Polymerization

**Figure 7:**
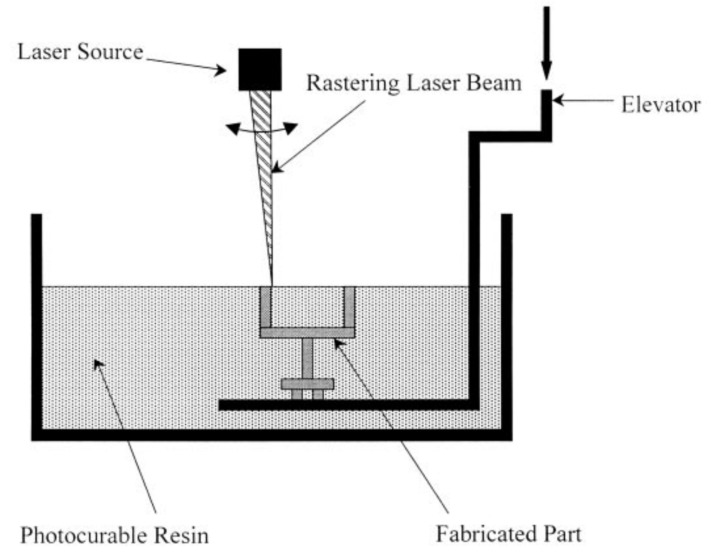
Schematic of the vat polymerization process [35]; with permission from Elsevier.

A schematic of the vat polymerization (VP) process is shown in Figure 7 [35]. VP involves the UV-assisted photopolymerization of liquid monomers. An UV laser is scanned over a layer of the liquid monomer to cure it in selected areas dictated by the tool paths. After the completion of one layer, another layer of resin is coated atop the cured layer. This process is called recoating. The process of recoating and curing is repeated until the part is completed [35].

### 2.8. Color 3D Printing Technology

Color 3D printing is a remarkable technology for customized manufacturing and integrated production in different industries, despite a few key issues such as printing speed and size for industrialization. Based on the printed substrates, color 3D printing techniques can be classified into six major categories: (a) plastic-based, (b) paper-based, (c) powder-based, (d) organism-based, (e) food-based, and (f) metal-based. These six techniques vary from colorization materials to processes. However, their similarity has its basis in the general subtractive color theory, as recommended and standardized by the International Color Consortium (ICC) and Commission Internationale de L’Eclairage (CIE) [36]. It should be noted that these standardization procedures have been designed for 2D printing but not for 3D printing. Color reproduction and stability can assess the surface quality of 3D-printed color objects. However, the color quality evaluation procedures of color 3D printing techniques are few, and hence, a detailed general guide for managing the color process of color 3D printing is a necessity for providers and customers. The following tools are used to evaluate the quality of color 3D printing.

#### 2.8.1. Color Measurement of Color 3D Printing

Color measurement is an essential tool for the evaluation of the surface color quality of reproductions based on CIEXYZ tristimulus values and related CIELAB values. Further, standard 2D color measurement conditions were both (CIEXYZ tristimulus and CIELAB values) defined by the CIE. According to those conditions, the measured angle and used illumination source are the two key parameters to achieve accurate color measurement in printing graphics. For 3D color models, another dimensional color information better than 2D reproduction has been indicated, especially for the spatial color effect [37]. 

#### 2.8.2. Color Specification of Color 3D Printing

Color specification is not a new term for traditional graphics printing, but it is worthy for the peer color communication of color 3D printing. Certainly, 3D color specification is a useful tool for engineers and researchers in the rapid prototyping industry to understand 3D color properties or workflows and further develop comprehensive color control hardware or toolkits. For example, the color data format of the 2D digital description did not contain 3D visual attributes such as texture, gloss, opacity, transparency, etc. The AxF file published by the X-Rite team offered a good direction to exploit a more simplified tool describing material (3D) appearance [38]. 

#### 2.8.3. Color Reproduction of Color 3D Printing

Color reproduction is becoming increasingly important in color 3D printing applications. Currently, color reproduction is regarded as the most direct index for color quality evaluation. Similarly, color difference metrics such as the CIEDE94 and CIEDE00 metrics proposed in the 2D printing field were used for evaluating the color reproduction precision of the printed 3D model surface color. Based on one color difference metric, 2D color correction for textures can be implemented by combining the X-Rite Color checker with a polynomial regression approach [39]. 

## 3. Introduction to the Artificial Neural Network (ANN) Algorithm

Machine learning (ML) can be classified into two major classes: (a) supervised ML and (b) unsupervised ML. One of the easiest ways to identify between the two is to check whether or not the given dataset has labels on it. Therefore, ANN is identified as a type of supervised ML because the model communicates the outcome to the given inputs. ANN is appropriate for 3D printing processes, as there are well-defined inputs and outputs for this manufacturing method. ANN has robust assessing skills to represent intricate, extremely nonlinear associations between inputs and outputs. The ANN structure contains three types of layers: (a) an input layer, (b) a hidden layer, and (c) an output layer [40], and each layer contains neurons. Parameters in ANN are usually called “weights” and show the linking magnitudes between neurons in two adjacent layers. The numerical values of these weights are usually calculated by the iterative training of ANN to lessen the loss function between estimations and absolute output values. The most common method for the upgradation of weights is called back-propagation, which practices a chain rule to calculate gradients iteratively for each layer [41]. After training, ANN can conclude outputs based on the concealed input values. Three classes of ANN, given in Table 2, have proven their robustness and received popularity.

**Figure 8:**
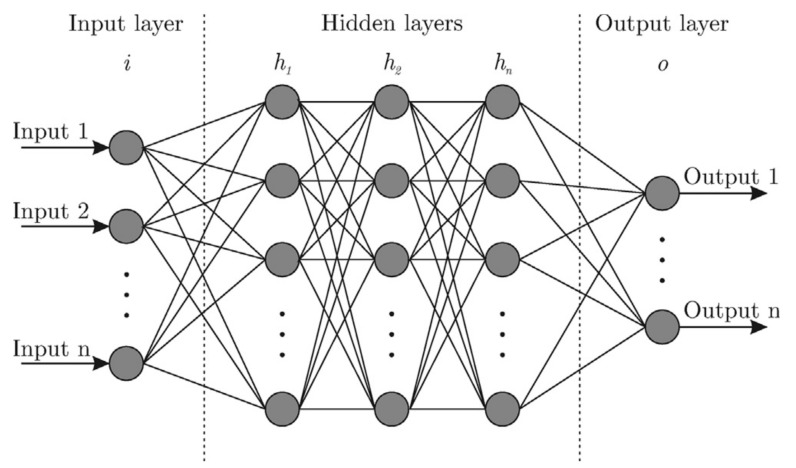
Structure of ANN [45]; with permission from Elsevier.

The structure in ANN typically consists of four significant subsections: (a) the number of hidden layers, (b) the number of neurons in each layer, (c) the activation function, and (d) the loss estimation function. A schematic diagram of the ANN is shown in Figure 8 [45]. 

Table 3 discusses the structure of an ANN in detail.

Based on the above information, the various ANN structures applied in the 3D printing processes are presented in Table 4.

## 4. Applications of ANN in 3D Printing

The 3D printing process combines several aspects, including the design of the model, selection of material, printing process, and manufactured part evaluation and characterization. This section combines the application of ANN for the 3D printing process in the case of procedure monitoring, designing, and the correlation between process parameters and final characteristics of the obtained component.

### 4.1. Process Monitoring

**Figure 9:**
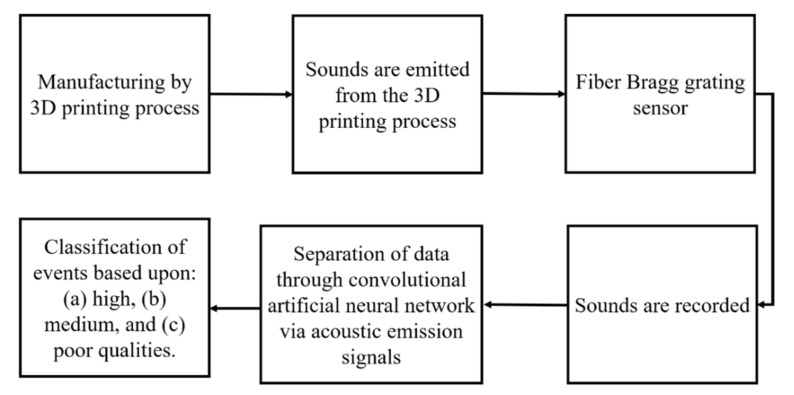
Schematic of the workflow used to monitor the quality during the printing process; based on the data from [73,74].

Process monitoring via various sensors, during the printing process, usually provides direct information w.r.t quality supervision and control. Three types of data sources can be identified as: (a) one-dimensional, such as spectra; (b) two-dimensional, such as graphs and images; and (c) three-dimensional, such as morphologies [72]. One-dimensional data can be processed faster but provide less information compared to two- and three-dimensional data. Shevchik et al. [73,74] investigated the possibility to use acoustic emission to monitor quality by combining the acoustic emission sensor with the ANN. Figure 9 shows a schematic of the workflow used to monitor the quality during the printing process.

**Figure 10:**
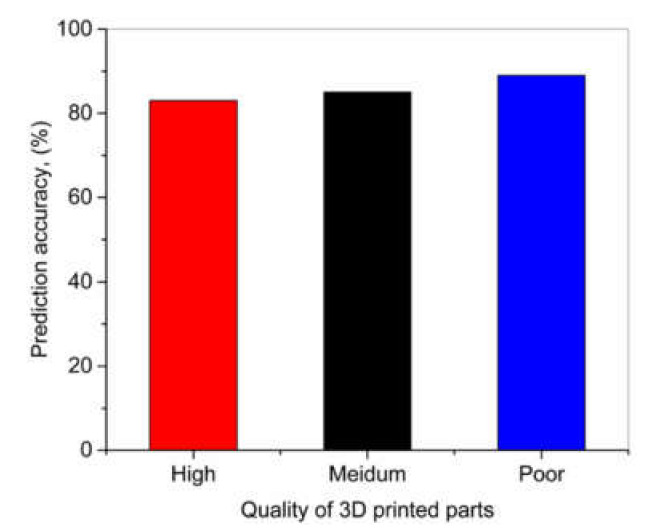
Prediction accuracy for high-, medium-, and poor-quality 3D-printed parts; based on the data from [73,74].

A fiber Bragg grating sensor was used to record the signals during the selective laser melting process. Low-, medium-, and best-quality parts were intentionally produced by process parameters tuning. The singles obtained were classified for training and testing. The convolutional ANN was used for this purpose. The inputs of this model were narrow frequency bands, while the outputs were the classification of the printed part of high, medium, or low quality. It was found that the convolutional ANN can give a result with accuracies of 83, 85, and 89% for high-, medium-, and poor-quality workpieces, respectively, as shown in Figure 10.

**Figure 11:**
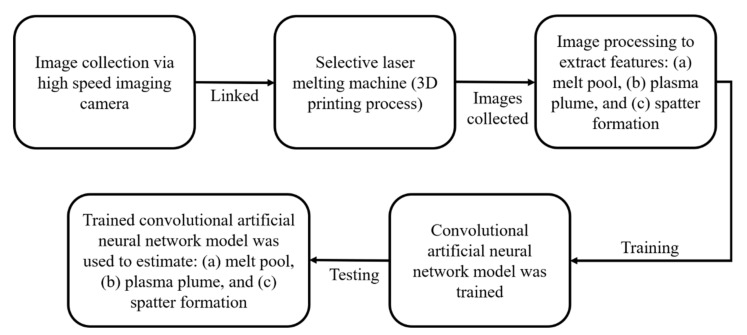
Schematic used by Zhang et al. [75] to train a convolutional ANN based on the image processing technique.

Zhang et al. [75] used a high-speed camera for the collection of process images. This system can also be called a visual system. This setup was able to collect three pieces of information: (a) melt-pool, (b) plasma plume, and (c) spatter formation. The features were sensibly taken out from the images after feeding them into a conventional ML algorithm. The algorithm proposed by the authors is presented in Figure 11. Further, the authors observed that even though the convolutional ANN model does not need a feature extraction step, it still can predict results with 92.7% accuracy. It makes convolutional ANN a potential candidate for real-time monitoring in the 3D printing process.

The detection of flaws through human-created, condition-based algorithms requires an in-depth understanding of the printing process as well as computer vision knowledge. Such condition-based algorithms are more restrictive; new algorithms have to be generated when new materials become available or when new part geometries are introduced, as this method needs to consider the interactions between various parameters. The reliance on the human operator makes condition-based algorithms less practical. ANN allows anomaly detection through a large dataset of good printing samples and bad printing samples, and the detection capability can be improved by adding new training data. As most in situ monitoring use cameras to acquire information about the printing condition, defect detection relies heavily on the capability of computer vision (CV). The most used ANN technique in computer vision is CANN, although other techniques have been used as well. For instance, Scime and Beuth [76,77] used scale-invariant feature transform (SIFT) to extract melt-pool features. They adopted various feature extraction techniques such as a bag of words (BOW) or histogram of oriented gradients (HOG) clustering to extract useful features from images and feature vectors. The feature vector was then fed to the SVM image classification algorithm to learn the defects, such as under-melting, key-holing, and balling [78]. They also attempted to use ANN techniques with CV to detect anomalies such as re-coater hopping and streaking, debris, superelevation, part failure, and incomplete powder spreading. Although the ANN algorithm can predict no anomalies with 100% accuracy, the algorithm was not able to predict re-coater streaking with high accuracy (50.6%). They compared the BOW technique with multiscale CANN (MCANN) and found that MCANN can achieve higher classification accuracies, though it is more computationally expensive (75% slower). Self-organizing error-driven ANN (SEANN), a combination of SOM and ANN, is found to be more accurate in classifying porosity defects than a k-nearest neighbor (KNN) algorithm and multilayer perceptron (MLP) [79]. Ye et al. [80] used a deep belief algorithm that consisted of stacking-restricted Boltzmann machines (RBMs), which had undirected connections between its top two layers and directed connections between all following adjacent layers, classifying the plume and spatter with minimum pre-processing and no feature extraction. The deep belief algorithm could achieve an 83.4% accuracy. In another work, to extract melt-pool, plume, and splatter data, CANN (92.7%) was found to have higher classification accuracy compared to SVM (89.6%) and the combination of SVM and principal component analysis (PCA) (90.1%) [75].

### 4.2. Designing

**Figure 12:**
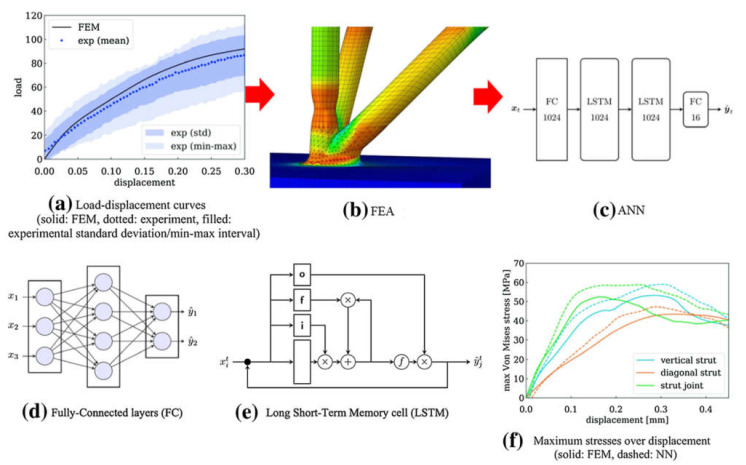
Overview of applying ANN for highly efficient numerical modeling [87]. (**a**) Experimental test to confirm the FE simulation results. (**b**) FE simulation results used as input for ANN. (**c**) ANN architecture containing one fully connected layer followed by two long-short-term memory (LSTM) cells, followed by another fully connected layer. (**d**) Schematics of fully connected layers. (**e**) Schematics of LSTM. (**f**) Comparison with FE simulation showing ANN capability in predicting stresses; with permission from Elsevier.

Design is an important research topic that requires a comprehensive understanding of the capabilities and limitations of 3D printing techniques. It is the first and critical step in the process workflow. A good CAD model design would not only ensure the printability but would also reduce the amount of support material when needed. However, the design process is normally iterative and time-consuming. A data-driven design for 3D printing would help designers in the design process. Maidin et al. [81] showed that the design feature database provided ideas and design features for less-experienced designers. The use of the ANN technique in 3D printing enables feature recommendations to existing CAD models, helping the designers to speed up the decision-making process during the design stage. For instance, Yao et al. [82] conceived a hybrid ANN algorithm, which used hierarchical clustering to classify 3D printing design features and a support vector machine (SVM) to enhance the hierarchical clustering results in the pursuit of the recommended design features. It helped inexperienced designers new to 3D printing to determine suitable design features for remote-controlled car components without actual physical trials and errors. Apart from that, ANN algorithms have been used for the feature recognition of CAD models and the manufacturability analysis of 3D printing. Heat kernel signature and the multiscale clustering method were used by Shi et al. to detect possible design faults in a particular CAD model [83]. A double-layered extreme ANN (DLEANN) was used by Zhang et al. to determine ideal print orientation to avoid putting support structures on user-preferred features [84]. In this DLEANN, the first layer was the classification to evaluate the relative score between the various part orientations, and the second layer was the regression to construct a global score for all printing directions. It was found to be able to identify the best printing directions with minimum visual artifacts due to support removal. Williams et al. [85] optimized the build orientation, and CANN was found to be better in terms of accuracy and consistency at predicting build time and part mass than the baseline linear regression model. Advancements in numerical simulations have allowed CAD models to be evaluated digitally before they are fabricated and tested physically, reducing the cost and time spent in experiments. However, numerical simulations can be computationally costly and time-consuming with complex processes, making online monitoring of the printing processes not feasible. Data-driven models have potential in predicting the final properties of the printed parts. Khadilkar et al. [86] used a deep-learning-based (DL) ANN framework to estimate stress distribution on the cured layer from selective laser sintering in almost real-time. In this attempt, a 3D model database that contained a wide range of geometric features was firstly generated. FE simulations on the 16,700 3D-printed models were then used to generate data labels to train the DL-ANN. They found that a two-stream CANN outperforms a single-stream CANN and ANN. Despite this, Koeppe et al. [87] used ANN to learn a parameterized mechanical model of cellular lattice structures, which includes their linear elastoplastic mechanical behavior to predict maximum von Mises and equivalent principal stresses in the struts and joints (Figure 12). The data-driven stress prediction took about 0.47 s, which was significantly shorter in comparison with the FE simulation, which took 5–10 h. The trained ANN models can potentially be incorporated into existing FE simulation frameworks to simulate the structural performance of larger parts of various scales. 

Chowdhury and Sam [88] explained that ANN algorithms could learn the thermal deformation of 3D printing processes and provide appropriate geometric compensation to the models for printing. Meng et al. [89] used 3D printing for the development of new designs, such as biomimetic structures. In particular, composite structures could now be tuned rapidly. ANN algorithms were demonstrated to be suitable for such areas, especially in tuning material properties and the capability to generate new designs that outperform existing composites available in the dataset by [90,91]. CANN was used to predict the stiffness and toughness of the composite. ANN simulation, which includes the training (*n* = 80,000) and predictive (*n* = 20,000) phases, is found to be 250 times quicker compared to FE simulations. It is shown that a small amount of training data is sufficient to obtain an ANN model with high accuracy. Furthermore, obtaining an optimal design for the composite remains possible with incomplete information. 

Table 5 provides a summary of research works on ANNs in design for 3D printing.

### 4.3. Correlation between Process Parameters’ and Parts’ Final Characteristics

In 3D printing, process parameter optimization is indispensable. Therefore, it is desirable to develop a direct correlation between process parameters and 3D-printed parts’ characteristics. However, this correlation is highly nonlinear as there are various input parameters involved in the 3D printing process, and it is tough and time-consuming to correlate all the factors mathematically. In this scenario, ANN can be used owing to its intrinsic nonlinear nature. Table 6 compiles the implementation of the ANN in 3D printing processes. It can be observed that various operating parameters were selected for different outputs, defining a deterministic relation between input and output for process optimization.

**Figure 13:**
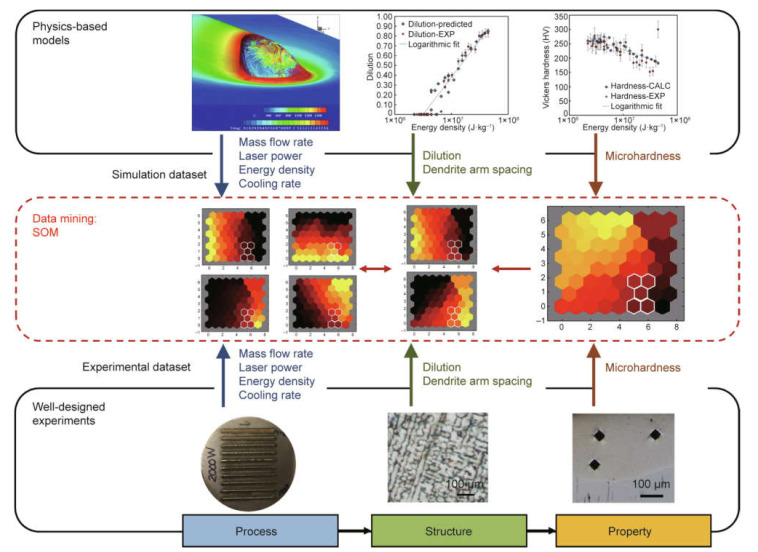
An illustration of the workflow normally used in current numerical studies (**top row**) and experimental studies (**bottom row**), accompanied by a description of how the ANN technique can be incorporated to discover useful process–structure–property relationships of certain materials [93]; published under the open access Creative Commons license with Elsevier.

Process parameters affect the 3D-printed parts’ properties [97,98,99]. A database of process–structure–property (PSP) relationships for a particular 3D printing process and materials would enable the proper selection of the parameters based on the available information in the database. The PSP relationship is often complicated due to a large number of process parameters, making it difficult to establish the governing mathematical formula of the process. Due to its complex nature, ANN algorithms have been used to determine the PSP relationships for many AM techniques. Gan et al. [93] attempted to use a self-organizing map (SOM) to identify the PSP relationship of the directed energy deposition process for Inconel 718. Multiple objective optimizations of the process parameters can be achieved from the large and high-dimensional dataset, which is obtained from simulation and validated with experimental results, with the help of visualized SOM, as shown in Figure 13. 

ANN is most commonly used for process optimization. Sarlo and Tarazaga [95] used ANN to design 3D-printed surrogate systems that match the dynamic characteristic of a target whose physical characteristics are not available. A total of 7500 random thickness profiles of beams were generated to train the ANN model to predict the suitable thickness profile of the beam for a certain frequency or mode shape. It is found that the ANN algorithm can predict surrogates with low modal error (<18%). A three-layer ANN structure is sufficient for process optimization, with the first layer being the input layer, the second being the hidden layer, and the third being the output layer. The number of neurons in the first layer depends on the number of input process parameters of the study. The number of neurons in the third layer is determined by the number of properties to be optimized, which is typically one or two. The number of neurons in the hidden layer is normally more than that of the input layer. The number of neurons in the hidden layer must be appropriate to avoid overfitting or underfitting issues in ML. Overfitting occurs when the noise in the training data is captured and learned as concepts by the model. In contrast, underfitting refers to the lack of fit of the model to the training data, which means that a good relationship between the data and the model is not obtained. Normalization of the input parameters is essential before they are used for ML models as it helps the ANN to learn faster and ensure the inputs are in an incomparable range. If the inputs are of different scales, the weights linked to some inputs will be updated much faster than others, which is undesirable. Hence, they are usually linearly normalized to be in the range of either [0, 1] or [−1, 1], using [97,98,99]:

(6) $$\frac{r_{i}-r_{min}}{r_{max}-r_{min}}$$

(7) $$\frac{2r_{i}-r_{min}}{r_{max}-r_{min}}$$

where *r_i_* is the particular input data, *r_min_* is the smallest input data, and *r_max_* is the largest input data, respectively. Various studies have compared ANN algorithms with conventional optimization methods such as the Taguchi method [53,100,101,102], polynomial regression model [67,103,104], and ANOVA [46,102,105]. Xiong et al. [103] predicted bead geometry during single-track melting using laser melting. A 4-12-2 ANN was found to achieve a lower mean of errors, registering 1.922% and 2.104% compared to a second-order regression model with a mean of errors of 2.633% and 2.308% for bead width and bead height predictions. In another study [104], ANN was found to have a better predictive ability for the dynamic modulus of elasticity of 3D-printed parts by achieving a higher R_2_ value and lower absolute average deviation compared to the fractional factorial model (despite having limited numbers of experiments). A 5-8-1 ANN model to predict the wear characteristics [100,101] was able to achieve a higher correlation coefficient (R_2_ value) of 0.9902 in comparison to the regression model’s 0.9516. The ability of ANN models to capture the nonlinearity between the input and output parameters has allowed complex 3D printing process mathematical models to be determined with higher accuracy. Table 7 summarizes the use of ANN algorithms in 3D process optimization, the input process parameters, and the target properties.

### 4.4. ANN for Metals’ and Polymers’ 3D Printing

Tak et al. [117] 3D printed a W-band slotted waveguide array antenna (SWAA). The SWAA consisted of three different sections (two horizontal and one vertical) such as a radiating waveguide array with 10 × 10 slots, an array with an aperture size of 31 mm × 31.4 mm, a coupling waveguide to feed the radiating waveguide array, and a vertical waveguide to feed the coupling waveguide. A machine-learning technique based on an ANN algorithm was used to optimize the design. The optimized SWAA was printed using stereolithography and then metalized with silver on the inner and outer surfaces by metal jet spraying. Non-radiating slots were added on the surface of the designed SWAA to metalize the inner and outer surfaces of the monolithic structure. The surface roughness was taken into account by employing the Huray model in the simulation. The manufactured SWAA showed a 22.5 dBi far-field gain, a −13.5 dB sidelobe level, and a 10° half-power beamwidth (HPBW) at 78.7 GHz during working. Wang et al. [118] presented a closed-loop control framework by seamlessly integrating the vision-based technique and ANN tool to inspect droplet behaviors and accordingly stabilize the liquid metal printing process (LMJP). This system automatically tuned the drive voltage applied to compensate for the uncertain influence based on vision inspection results. To this purpose, multiple features and properties from images to capture the droplet behavior were extracted. Further, an ANN, together with a PID control process, was used to determine the drive voltage. This system was tested on a piezoelectric-based ink-jetting emulator, which has a very similar jetting mechanism to the LMJP. Results showed that significantly more stable jetting behavior could be obtained in real time. This system can also be applied to other droplet-related applications, owing to its universally applicable characteristics. Laser power influences the formation of pores and cracks and determines the quality and density of a part. Kwon et al. [119] applied a deep ANN model to the selective laser melting process to study the classification model based on the melt-pool images for six laser power labels. Using an ANN in which the number of nodes is decreased while increasing the layer number achieved satisfactory inference when melt-pool images had blurred edges. The proposed ANN showed a classification failure rate under 1.1% for 13,200 test images. It was more effective to monitor melt-pool images because it simultaneously handled various shapes, compared with a simple calculation such as the sum of pixel intensity in melt-pool images. The classification model can be utilized to infer the location where unexpected alteration of microstructures or particular defective products can occur nondestructively.

Pant et al. [120] optimized the process parameters of fused deposition modeling (FDM) by exploring the wear performance of PLA. A variation of process parameters, such as layer thickness, orientation, and extruder temperature, was investigated. Based on these parameters, a wear specimen (according to ASTM G99) was printed using FDM. The wear behavior of the polymer pin under low sliding speed was examined. The Taguchi design of experiments by using an L_9_ orthogonal array was applied to optimize the process parameters at which the minimum wear rate was obtained; the same has also been investigated by using analysis of variance (ANOVA) and ANN technique for rigorous validation/optimization. The results show that this specific build orientation has a major influence on the wear performance of the polymer pin. The ANN was found to be limited due to the lack of datasets. To find the best processing parameters for minimizing warpage in the case of 3D printing of polymers by selective laser sintering, Dastjerdi et al. [121] presented an optimization algorithm. The FE method was used to simulate the sintering of a layer of polymer powder, and the warpage of the layer was calculated. The numerical model was verified by comparison with experimental results. A back-propagation ANN was used to formulate the mapping of the design variables and objective function. The results of 40 simulations with various input parameters such as scanning pattern and speed, laser power, the surrounding temperature, and layer thickness were used to train and test the ANN. An optimal pattern of scan and process parameters could be obtained using the optimization approach resulting in minimum warpage in the sintered layer. Fountas et al. [122] carried out an experimental study to examine the effect of five fused deposition modeling (FDM) parameters using PLA material such as layer height, shell thickness, infill density, orientation angle, and printing speed on the tensile strength of standard ASTM 638-10 type 1 tensile specimens. The experimental study involved a fractional factorial design with 16 runs. This design was then converted into a custom response surface design to examine the nonlinearity presented by the curvature when examining independent variables in continuous form. The study [122] not only gives an insight concerning the complex dependency of tensile load by the process parameters corresponding to FDM but also generates a statistically validated regression model. The regression model adequately explains the variation and the nonlinear influence of FDM parameters on tensile strength. Thus, it can be implemented to find optimal parameter settings with the use of any ANN algorithm.

The evaluation of AM components is often conducted using experiments assessing product quality, build time, dimensional accuracy and tolerances, production cost, and the tribological properties of parts. As it occurs in any other manufacturing process, the performance of AM is strongly affected by its corresponding process parameters. Fountas et al. [123] studied the performance of different swarm-based evolutionary algorithms regarding single- and multi-objective optimization problems related to the additive manufacturing of acrylonitrile butadiene styrene (ABS) with emphasis on FDM processes. Five problems were questioned regarding their number of independent variables and predetermined optimization objectives. Two of these problems were of a single objective, while three were of a multi-objective optimization nature. The results obtained by several independent executions of algorithms were compared by analogous indicators depending on the problem, i.e., convergence speed for the single-objective problem and quality of Pareto nondominated solutions in the case of multi-objective optimization problems. The algorithms tested for single-objective optimization were: the dragonfly algorithm (DA), the ant-lion algorithm (ALO), the grey-wolf algorithm (GWO), the moth-flame algorithm (MFO), and the whale optimization algorithm (WOA). For the multi-objective optimization problems, the multi-objective grey-wolf (MOGWO), the multi-objective ant-lion (MOALO), the multiverse algorithm (MOMVO), the multi-objective dragonfly (MODA), the Pareto envelope-based selection algorithm (PESA-II), and the strength Pareto evolutionary algorithm (SPEA-II) were tested. Even though all algorithms proved their capability of providing optimal solutions to cope with volatile scenarios, the “No-Free Lunch” theorem was validated, supporting that algorithms did not perform the same when applied to different optimization problems. 

## 5. Potential Challenges for the Implementation of ANN and Their Solutions

The following potential challenges have been identified based on the literature survey.

### 5.1. Datasets Optimization

The performance of an ANN model depends on the quantity and type of data provided while training. On the other hand, it is expensive and time-consuming to collect and organize the data for the training of an ANN model. Certain available methods can artificially enlarge a dataset, one of which is to use an artificial generative model [124]. It was explained that an autoencoder could randomly generate new datasets by keeping in view the training data [125]. The extension of this autoencoder is known as a variational autoencoder. Other generative models, including generative adversarial nets and adversarial autoencoders, can provide ways to carry out data augmentation [126,127].

### 5.2. Selection of Significant Input Parameters

The training of an ANN model majorly depends on the input parameters, while the operating parameters play a significant role in any process. A selection of an excessive number of input parameters can simply overfit an ANN model. Hence, it is essential to confirm that the ANN model is trained to function at an optimum number of input parameters. As illustrated in Table 8, there are two techniques available for the preprocessing of given data to determine the significant input parameters.

### 5.3. Under- and Over-fitting in the ANN Model

The main objective of an ANN model is a good estimation of the output based on previously given input data. This performance, however, can be disturbed due to the over- or under-fitting of an ANN model based on inputs. An overfitted ANN algorithm means that a model attempts to fit itself on every data point within the given dataset, and the model becomes susceptible to noises within the dataset. On the contrary, an underfitted model means that the model has failed to build up the mandatory relationships among the data points in the training dataset. These difficulties can be avoided by selecting the optimum number of neurons within each layer [129,130].

### 5.4. Linking the Analytical Modeling and Numerical Simulations with ANN

To avoid a significant number of experimentations and produce a pool of datasets for the optimization of the ANN model, it is recommended to develop analytical or numerical simulations. The results of analytical simulations can be used to train the ANN models. Methodology that combined the experiments, FE simulations, and ANN is given in [77]. Initially, experimental analyses were conducted to validate FE simulations. Later, 85 simulation analyses were carried out using various parametric combinations, such as load, displacement, strut radius, and cell scale (input). At the same time, the maximum von Mises and principal stresses were categorized as outputs. After training was conducted for the ANN model, and a close correlation for the loading history was found between ANN estimations and FE simulations [88].

### 5.5. Real-Time Monitoring of the 3D Printing Process

**Figure 14:**
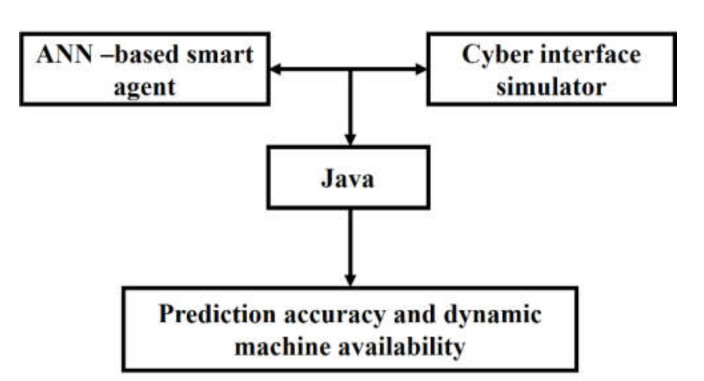
Dynamic 3D printing machine identification system.

The final properties of a developed part are defined by layer-by-layer deposition. Therefore, it is important to monitor and control the quality of the deposited layer in real time. To this purpose, a dynamic machine identification system can be developed by integrating the outputs from both the ANN-based smart agent and the cyber interface simulator to compute the final machine assignment, as shown in Figure 14.

The following steps are to be undertaken to translate this prototype system (Figure 14) into full-scale implementation:

(1) Collection of a larger number of part designs, based on different variety of geometries, topologies, and material types;
(2) Increasing and/or altering the input variables/factors for evaluating the part designs based on the application requirement. However, these factors may be updated based on user requirements;
(3) Developing software with a graphical user interface (GUI) that can integrate the different subsystems. It can assist both novice users in inputting their datasets as well as 3D printing specialists to modify the ANN algorithm parameters to tune their results; and
(4) Collaboration with industry partners so that a joint system specific to each industry sector (such as automotive, aerospace, consumer appliances, etc.) be developed.

## 6. Future Outlook

For data-driven ANN, the preprocessing of data is a crucial prerequisite, as it erases the incorrect data and uses the filtered data to train an ANN. However, this step requires tedious/laborious/meticulous work. For instance, if an ANN model requires only cracks as input from the scanning electron images of deposited layers, there will be a need to filter out the data, accordingly. However, the difficulty is to extract cracks’ locations precisely, distributed along the grain peripheries. It is a challenging task for those who do not have reliable information and practice in image processing to extract the exact information. Therefore, there is a strong need to develop standards and practices for data preprocessing. There are various databases, such as MatWeb [131], available to store the material properties electronically. Due to the high complexities and varieties in the field of 3D printing, it is essential to develop a new database to deal with the huge number of results achieved from different research groups.

As discussed earlier in Section 4.1, scientists have developed various systems to deliver real-time statistics in the 3D printing process. To this purpose, various sensors have been used to perceive and quantify data. However, there remains a need for development in this area. For instance, the sensing devices used in the 3D printing process must survive under harsh working environments. In the laser-melting deposition process, the temperature intensity is too high, and the plasma plume formation can destroy the imaging camera lens. Further, the sensors should be fast enough to measure the melt-pool dimensions. To this purpose, reliable systems should be developed. Usually, dedicated software is needed to control such sensors. The software should be capable of monitoring, capturing, processing, and storing the data. Therefore, during the printing process, there is a need to develop software with machine-learning algorithms to compute thermal profiles, extract melt-pool dimensions, identify microstructure, and detect voids and porosity.

The 3D printing process utilizes layer-by-layer deposition; hence, the quality of each layer countlessly impacts the final part’s characteristics. Therefore, it is highly desirable to control the quality of parts at the layer level. Sensors using electrical, thermal, and optical signals can provide in situ monitoring of the 3D process. A possible solution is to use a convolutional ANN model in combination with a high-speed imaging camera to obtain quality feedback at each layer, resulting in a closed-loop system speed camera. In this case, the ANN algorithm must quickly respond to the input picture.

Multiphysics-based models require considerable computation time and cost. As explained above, it is possible to link numerical simulations with ANN models. After validating the numerical simulation with experimental data, the numerical models can be used to train ANN models, which in return, can be utilized for forecasting based on operating parameters.

The authors believe that after addressing the areas mentioned above, the implementation of ANN can be increased in the field of 3D printing.

## 7. Conclusions

In the present era, 3D printing and ANN have gained popularity. Three-dimensional printing has several advantages over traditional manufacturing technologies. Recently, it has proved its capability to manufacture complex morphologies. On the other hand, ANN models avoid creating and cracking complex multi-physics models. Therefore, the combination of 3D printing and ANN has demonstrated great potential for achieving the concept of “Industry 4.0”. Herein, the structure of ANN, its implementation in the field of 3D printing, potential challenges while implementing ANN, and future trends have been presented, described, and discussed. From this study, one can conclude that:

- ANN involves supervised learning primarily composed of three layers: (a) an input layer, (b) a hidden layer, and (c) an output layer. Three classes, including multilayer perceptron, convolutional ANN, and recurrent ANN, have been found. The ANN structure contains four hyperparameters: (a) the number of the hidden layers, (b) neurons, (c) the activation function, and (d) the loss function. Two types of error functions have been identified in the case of the 3D printing process: (a) Tanh and (b) Sigmoid.
- ANN can be used for product designing, process monitoring, and to correlate the input parameters with the properties of the final produced part. In the 3D printing process, it is very tough to optimize operating parameters, as they are highly nonlinear in nature. This task, however, can efficiently be completed using an ANN model, owing to its nonlinear nature. In this context, convolutional ANN has proved capabilities to forecast with better precision compared to other classes of ANN. According to Table 4, 5–10 neurons in the hidden layer are suggested to determine the optimal solution in the 3D printing process.
- The performance of an ANN model depends on the quantity and type of data provided while training. Further, it is expensive and time-consuming to collect and organize the data for the training of an ANN model. Therefore, it is necessary to determine the significant set of parameters to save time and train an ANN model, effectively. It will also avoid the over- or underfitting of the ANN model. On the other, artificial datasets can be generated via analytical or numerical modeling to avoid the deficiency of datasets needed for ANN training and testing.

## Figures and Tables

**Table 1:** Various 3D printing processes [24].

Sr. No.	3D Printing Processes	Process Illustration
1	Binder jetting	A liquid bonding agent is selectively poured to join powder materials
2	Direct energy deposition	Focused thermal energy (laser, electron, or plasma arc) is used to fuse materials by melting as they are being deposited
3	Material extrusion	Material is selectively dispensed through a nozzle or orifice
4	Material jetting	Droplets of building material are selectively deposited
5	Powder bed fusion	Thermal energy selectively fuses regions of a powder bed
6	Sheet lamination	Sheets of material are bonded to form a part
7	Vat photopolymerization	Liquid photopolymer in a vat is selectively cured by light-activated polymerization

**Table 2:** Three classes of ANN models.

**Multilayer Perceptron**	The most typical ANN, common in linear and nonlinear summations, e.g., sigmoid functions. It is frequently used for the data in the tabular [42].
**Convolutional ANN (CANN)**	Deliberates the relationship between image pixels and is therefore used in image processing [43].
**Recurrent ANN**	Has a significant role in temporal dynamics, as it can build a connection between the number of nodes in a given layer. Therefore, it is used in long short-term memory that can regenerate the simulation results accurately [44].

**Table 3:** Structure of an ANN model.

**No. of hidden layers**	A combination of the input-hidden-output layer with a respective number of neurons is used to define the ANN model [46]. For instance, 5-8-1 means that the input layer has 5 neurons, 8 neurons in the hidden layer, while the output layer has only 1 neuron.	
**No. of neurons in a given layer**	The required number of neurons in the input and output layer depends on the particular problem. However, the number of neurons must be chosen carefully. If the number of neurons is not selected optimally, it will lead to under- or overfitting in the given dataset [47]. Various studies have been carried out that suggest choosing a number of neurons between 5 and 10 [48,49,50,51,52,53,54,55,56,57].	
**Activation function**	A nonlinear transformation on a given input signal. In other words, it decides whether to activate and deactivate a particular neuron. Based on its performance, the activation function is considered a vital part of an ANN model. It is important to mention that a network deprived of an activation function behaves like a linear regression model, which cannot deal with the complicated tasks [58]. A few activation functions are:	
	$\mathrm{ReLU}\;\left(x\right)\;=\max\left(0,x\right)$	(1)
	$\mathrm{Tanh}\left(x\right)\;=\frac{2}{1-e^{-2x}}-1$	(2)
	$\mathrm{Sigmoid}\;\left(x\right)\;=\frac{1}{1-e^{-x}}$	(3)
**Loss function**	The loss function is usually determined using real-world problems and carries the interpretation of real-time data. The root means square (RMS) and the absolute mean error (AME) are two commonly used methods to estimate the difference between the predicted vector and the target value [59]. Their mathematical expressions are given as:	
	$RMS=\;\sqrt{\frac{\sum_{i=1}^{n}{\left(x_{i}-x_{t}\right)}^{2}}{n}}$	(4)
	$AME=\frac{\sum_{i=1}^{n}\left|x_{i}-x_{t}\right|}{n}$	(5)
	where *i* is a sample index, *x_i_* is a predicted value, and *x_t_* is the target value.	

**Table 4:** ANN structure in various 3D printing processes.

3D Printing Process	Input-Hidden-Output Layers	Activation Function	Error Between Absolute Output and Anticipated Output (%)	References
Selective laser sintering	4-9-1	Sigmoid	6.99	[60]
	4-6-1		1.06	[61]
	3-7-1		15.01	[62]
	7-7-1		4.36	[63]
	5-27-1		0.91	[64]
	7-8-1		0.90	[64]
	3-9-1	Tanh	0.50	[56]
Stereolithography	6-20-5	Sigmoid	5.98	[65]
Laser-melting deposition	3-9-3	Sigmoid	3.0	[66]
Fused deposition modeling	5-8-1	Tanh	1.99	[46]
	5-8-1		1.02	[67]
	4-15-12-1	Sigmoid	5.2	[68]
	5-6-4		4.08	[69]
	5-7-3		0.11	[70]
Binder jetting	4-20-1	Sigmoid	0.40	[71]

**Table 5:** ANN in various 3D printing processes.

Features	ANN Technique	Remarks	References
Composite design	Linear model and CANN	Predict mechanical properties accurately even with a small amount of training data Ability to rebuild detailed performances of designs without using precise information in the training process	[90,91]
Process planning	Genetic algorithm (GA) and classical gradient-based schemes	Included design search space restrictions, which make the objective function not continuously differentiable in design space Highly nonconvex	[92]
Design feature recommendation	Hierarchical clustering and SVM	Assist novice designers in discovering AM-enabled design freedoms Only performance-centric design knowledge (i.e., “loadings”, “objectives” and “properties”) has been considered in 3D printing design feature recommendation	[82]
Tuning microstructure and microhardness	Self-organizing map	Included physics-based models, experimental measurements, and a data-mining method Dendrite arm spacing and microhardness are approximated using the mechanistic models	[93]
Optimize build orientation concerning build time and part mass	10-layer CANN and linear Regression model	CANNs are most precise at estimating all three studied factors than the baseline linear regression model for the training and evaluation conditions explored	[85]
Flatness perception	Classification tree (C4.5)	The results indicated some differences in the perception of fatness quality	[94]
Geometric compensation	Feedforward ANN	Uses the FE model to simulate the deformations in the 3D-printed part Geometrical compensation is performed on the. STL file of the part using the trained network	[88]
Part orientation	DLEANN	Used to assess part orientation based on viewpoint preference, visual saliency, smoothness entropy, and area of support Scores of a part printed in different orientations are assessed	[84]
Designing surrogate systems	ANN	7500 random thickness beams and corresponding FE solutions are generated to train the ANN Able to replicate the dynamic characteristic of a target whose physical characteristics are inaccessible or unknown	[95]
Composite design	CANN	Used ANN for coarse-graining analysis and designing materials without the use of full microstructural data Coarse graining is achieved by condensing a group of building blocks into a single unit cell, which greatly lowers the number of parameters required in the ANN algorithm	[90,91]
Stress prediction	2-Stream CANN	16,700 models of data labels are created using FE simulation Parameters such as peak stress and dependence on previous layer information are investigated The deep learning model outperforms the simple neural network model used for stress prediction	[86]

**Table 6:** Applications of an ANN in the field of 3D printing.

3D Printing Process	ANN Input Parameters	ANN Output Parameters	References
**Selective laser sintering**	Laser power, scanning speed, hatch spacing, powder layer thickness	Density	[60]
	Laser power, scanning speed, hatch spacing, powder layer thickness	Geometrical dimensions	[61]
	Vertical height, deposited volume, bounding box	Manufacturing time	[62]
	Laser power, scanning speed, hatch distance, powder layer thickness, scanning mode, temperature distribution, the processing time	Shrinkage percentage	[63]
	Powder layer thickness, laser power, scanning speed	Part porosity	[64]
	Laser power, scanning speed, hatch distance, powder layer thickness, temperature distribution	Tensile strength	[64]
	Laser power, scanning speed, hatch distance, powder layer thickness, scanning mode, temperature distribution, the processing time	Density	[96]
**Stereolithography**	Powder layer thickness, curation time, hatch distance, filling cure depth, filling spacing depth	Geometrical dimensions (precision)	[65]
**Laser-melting deposition**	Laser power, scanning speed, powder feed rate	Geometrical dimensions (precision)	[66]
**Fused deposition modeling**	Layer thickness, positioning, raster angle and width, air gap	Compressive strength	[46]
	Layer thickness, positioning, raster angle and width, air gap	Wear	[67]
	Positioning, slice width	Deposition error in volume	[68]
	Layer thickness, positioning, raster angle and width, air gap	Dimensional precision	[69,70]
**Binder jetting**	Layer thickness, printing saturation, heater power ration, drying time	Surface roughness, shrinkage	[71]

**Table 7:** The use of an ANN for process optimization of 3D processes.

Process	Purpose	ANN Method	Input Parameters	References
**Binder Jetting**	Predicting surface roughness, shrinkage rate in *y*- and *z*-directions	3-layer BP-ANN	Layer thickness, printing saturation, heater power ratio, drying time	[53]
	Compressive strength, open porosity	Aggregated ANN	Orientation, layer thickness, delay time	[106]
	To characterize defects evolution	Gaussian mixture model	To reduce pore decomposition, shrinkage and smoothing during post-processing	[107]
**Selective laser sintering**	Dimension	Radial basic function ANN, fuzzy C-means, and pseudoinverse method, k-means	Laser power, scan speed, scan spacing, layer thickness	[48]
	Material analysis	ANN	Structural characterization	[108]
	Shrinkage ratio	ANN	Laser power, scan speed, hatch spacing, layer thickness, scan mode, temperature, interval time	[55]
	Tensile strength	ANN	Laser power, scan speed, hatch spacing, layer thickness, powder temperature	[109]
	Density	ANN	Laser power, scan speed, hatch spacing, layer thickness, scan mode, temperature, interval time	[110]
**Selective laser melting**	Keyhole porosity	K-means clustering	Energy density	[111]
**Stereolithography**	Dimensional accuracy	ANN	Layer thickness, border overcure, hatch overcure, fill cure depth, fill spacing, and hatch spacing	[112]
	Printability	Ensemble method, Siamese network	Printability	[113]
**Laser-melting deposition**	Geometrical accuracy	ANN	Laser power, scanning speed, powder feeding rate	[114]
	Melt-pool width	ANN	Laser power, powder feed rate, laser speed, focal length, contact tip to workpiece distance	[115]
**Electron beam melting**	Volume, roughness	ANN	Spreader translation speed, rotation speed	[116]

**Table 8:** Techniques for preprocessing of a given dataset [59,128].

**Feature Selection**	**Illustration**
This technique assists in determining the most influencing parameters from a given list using statistical tools.	To determine the parameter significantly affecting the printing process, a Pearson’s coefficient can be determined to figure out the dependency between the given parameters on output. If Pearson’s value (max = 1) is higher for one parameter compared to the other parameter, it will affect the desired output significantly.
**Feature Combination**	**Illustration**
This technique helps to carry out dimensionality lessening for input attributes and thereby concentrate on the newly generated features. Once the translation regulation is identified, manual manipulations are usually preferred. Mathematical tools such as principal components analysis can be utilized for the same purpose based on the attribute.	Energy density (ED) influences the solidification, metallurgical, microstructure, and mechanical properties of a 3D-printed part. Laser power, scanning speed, hatch distance, and layer thickness combine and generate a new ED feature.

## Data Availability

Not applicable.

## References

[1] Lu S., Zhou L.N., Wang Z. (2015). Damage Evolution of Concrete by Electrical Resistivity Monitoring Methods. Appl. Mech. Mater..

[2] Derby B. (2015). Additive Manufacture of Ceramics Components by Inkjet Printing. Engineering.

[3] Gu D., Ma C., Xia M., Dai D., Shi Q. (2017). A Multiscale Understanding of the Thermodynamic and Kinetic Mechanisms of Laser Additive Manufacturing. Engineering.

[4] Herzog D., Seyda V., Wycisk E., Emmelmann C. (2016). Additive manufacturing of metals. Acta Mater..

[5] Liu L., Ding Q., Zhong Y., Zou J., Wu J., Chiu Y.L., Li J., Zhang Z., Yu Q., Shen Z. (2018). Dislocation network in additive manufactured steel breaks strength–ductility trade-off. Mater. Today.

[6] Mahmood M.A., Popescu A.C., Hapenciuc C.L., Ristoscu C., Visan A.I., Oane M., Mihailescu I.N. (2020). Estimation of clad geometry and corresponding residual stress distribution in laser melting deposition: Analytical modeling and experimental correlations. Int. J. Adv. Manuf. Technol..

[7] Acharya R., Sharon J.A., Staroselsky A. (2017). Prediction of microstructure in laser powder bed fusion process. Acta Mater..

[8] Fergani O., Berto F., Welo T., Liang S.Y. (2017). Analytical modelling of residual stress in additive manufacturing. Fatigue Fract. Eng. Mater. Struct..

[9] Chen Q., Guillemot G., Gandin C.A., Bellet M. (2017). Three-dimensional finite element thermomechanical modeling of additive manufacturing by selective laser melting for ceramic materials. Addit. Manuf..

[10] Wang Y., Li X. (2020). An accurate finite element approach for programming 4D-printed self-morphing structures produced by fused deposition modeling. Mech. Mater..

[11] Wu X., Xu C., Zhang Z., Guo C. (2020). Modeling and visualization of layered curing conversion profile in ceramic mask projection stereolithography process. Ceram. Int..

[12] Zhang Y., Chou Y.K. (2006). Three-dimensional finite element analysis simulations of the fused deposition modelling process. Proc. Inst. Mech. Eng. Part B J. Eng. Manuf..

[13] Zhang Y., Chou Y.K. (2008). A parametric study of part distortions in fdm using 3d fea. Proc. Inst. Mech. Eng..

[14] Bellini A., Güçeri S., Bertoldi M. (2004). Liquefier dynamics in fused deposition. J. Manuf. Sci. Eng. Trans. ASME.

[15] Venkataraman N., Rangarajan S., Matthewson M.J., Harper B., Safari A., Danforth S.C., Wu G., Langrana N., Guceri S., Yardimci A. (2000). Feedstock material property—Process relationships in fused deposition of ceramics (FDC). Rapid Prototyp. J..

[16] Ju Y., Xie H., Zheng Z., Lu J., Mao L., Gao F., Peng R. (2014). Visualization of the complex structure and stress field inside rock by means of 3D printing technology. Chin. Sci. Bull..

[17] Sachs E., Vezzetti E. (2005). Numerical simulation of deposition process for a new 3DP printhead design. J. Mater. Process. Technol..

[18] Curodeau A. (1995). Three Dimensional Printing of Ceramic Molds with Accurate Surface Macro-Textures for Investment Casting of Orthopaedic Implants.

[19] Lecun Y., Bengio Y., Hinton G. (2015). Deep learning. Nature.

[20] Krizhevsky A., Sutskever I., Hinton G.E. ImageNet classification with deep convolutional neural networks. Proceedings of the Advances in Neural Information Processing Systems.

[21] Anusuya M., Katti S. (2010). Speech recognition by machine, a review. Int. J. Comput. Sci. Inf. Secur..

[22] Devlin J., Chang M., Lee K., Toutanova K. Bert: Pre-training of deep bidirectional transformers for language understanding. Proceedings of the NAACL-HLT 2019.

[23] Ondruska P., Posner I. Deep tracking: Seeing beyond seeing using recurrent neural networks. Proceedings of the AAAI-16 Conference.

[24] ISO/ASTM 52900:2015(en), Additive Manufacturing—General Principles—Terminology. https://www.iso.org/obp/ui/#iso:std:iso-astm:52900:ed-1:v1:en.

[25] Mahmood M.A., Popescu A.C., Mihailescu I.N. (2020). Metal Matrix Composites Synthesized by Laser-Melting Deposition: A Review. Materials.

[26] Mahmood M.A., Popescu A.C., Oane M., Ristoscu C., Chioibasu D., Mihai S., Mihailescu I.N. (2020). Three-Jet Powder Flow and Laser–Powder Interaction in Laser Melting Deposition: Modelling Versus Experimental Correlations. Metals.

[27] Mahmood M.A., Han C.F., Chu H.Y., Sun C.C., Wu W.H., Lin W.J., Liu L.C., Lai J.Y., Mihailescu I.N., Lin J.F. (2020). Effects of roll pattern and reduction ratio on optical characteristics of A1008 cold–rolled steel specimens: Analytical approach and experimental correlations. Int. J. Adv. Manuf. Technol..

[28] Mahmood M.A., Tsai T.Y., Hwu Y.J., Lin W.J., Liu L.C., Lai J.Y., Pan J.W., Li W.L., Lin J.F. (2020). Effect of fractal parameters on optical properties of cold rolled aluminum alloy strips with induced surface deflection: Simulations and experimental correlations. J. Mater. Process. Technol..

[29] Chioibasu D., Mihai S., Mahmood M.A., Lungu M., Porosnicu I., Sima A., Dobrea C., Tiseanu I., Popescu A.C. (2020). Use of X-ray Computed Tomography for Assessing Defects in Ti Grade 5 Parts Produced by Laser Melting Deposition. Metals.

[30] Ziaee M., Crane N.B. (2019). Binder jetting: A review of process, materials, and methods. Addit. Manuf..

[31] Sing S.L., Tey C.F., Tan J.H.K., Huang S., Yeong W.Y. (2019). 3D printing of metals in rapid prototyping of biomaterials: Techniques in additive manufacturing. Rapid Prototyping of Biomaterials: Techniques in Additive Manufacturing.

[32] Sidambe A. (2014). Biocompatibility of Advanced Manufactured Titanium Implants—A Review. Materials.

[33] Sireesha M., Lee J., Kranthi Kiran A.S., Babu V.J., Kee B.B.T., Ramakrishna S. (2018). A review on additive manufacturing and its way into the oil and gas industry. RSC Adv..

[34] Zhang Y., Jarosinski W., Jung Y.G., Zhang J. (2018). Additive manufacturing processes and equipment. Additive Manufacturing: Materials, Processes, Quantifications and Applications.

[35] Safari A., Allahverdi M. (2001). Electroceramics: Rapid Prototyping. Encyclopedia of Materials: Science and Technology.

[36] MacAdam D.L. (1938). Subtractive Color Mixture and Color Reproduction. J. Opt. Soc. Am..

[37] Brunton A., Arikan C.A., Tanksale T.M., Urban P. (2018). 3D printing spatially varying color and translucency. ACM Trans. Graph..

[38] Shishkovsky I. (2016). New Trends in 3D Printing.

[39] Chen C., Chen G.X., Yu Z.H., Wang Z.H. (2014). A new method for reproducing oil paintings based on 3D printing. Appl. Mech. Mater..

[40] Goldberg Y. (2017). Neural Network Methods for Natural Language Processing. Synth. Lect. Hum. Lang. Technol..

[41] Rumelhart D.E., Hinton G.E., Williams R.J. (1986). Learning representations by back-propagating errors. Nature.

[42] Gardner M.W., Dorling S.R. (1998). Artificial neural networks (the multilayer perceptron)—A review of applications in the atmospheric sciences. Atmos. Environ..

[43] Rawat W., Wang Z. (2017). Deep convolutional neural networks for image classification: A comprehensive review. Neural Comput..

[44] Mikolov T., Karafiát M., Burget L., Černocký J., Khudanpur S. (2010). Recurrent Neural Network Based Language Model. Proceedings of the 11th Annual Conference of the International Speech Communication Association.

[45] Bre F., Gimenez J.M., Fachinotti V.D. (2018). Prediction of wind pressure coefficients on building surfaces using artificial neural networks. Energy Build..

[46] Sood A.K., Ohdar R.K., Mahapatra S.S. (2012). Experimental investigation and empirical modelling of FDM process for compressive strength improvement. J. Adv. Res..

[47] Xu S., Chen L. A novel approach for determining the optimal number of hidden layer neurons for FNN’s and its application in data mining. Proceedings of the 5th International Conference on Information Technology and Applications.

[48] Staiano A., Tagliaferri R., Pedrycz W. (2006). Improving RBF networks performance in regression tasks by means of a supervised fuzzy clustering. Neurocomputing.

[49] Acharya U.R., Oh S.L., Hagiwara Y., Tan J.H., Adam M., Gertych A., Tan R.S. (2017). A deep convolutional neural network model to classify heartbeats. Comp. Biol. Med..

[50] Cheng H.D., Xu H. (2000). A novel fuzzy logic approach to contrast enhancement. Pattern Recogit..

[51] Wu S., Chow T.W.S. (2007). Self-organizing and self-evolving neurons: A new neural network for optimization. IEEE Trans. Neural Netw..

[52] Joseph J., Pignatiello J.R. (2007). An overview of the strategy and tactics of Taguchi. IIE Trans..

[53] Murphey Y.L., Guo H., Feldkamp L.A. (2004). Neural learning from unbalanced data. Appl. Integ..

[54] Zhang S.U. (2018). Degradation classification of 3D printing thermoplastics using Fourier transform infrared spectroscopy and artificial neural networks. Appl. Sci..

[55] Rong-Ji W., Xin-Hua L., Qing-Ding W., Lingling W. (2009). Optimizing process parameters for selective laser sintering based on neural network and genetic algorithm. Int. J. Adv. Manuf. Technol..

[56] Garg A., Tai K., Savalani M.M. (2014). State-of-the-art in empirical modelling of rapid prototyping processes. Rapid Prototyp. J..

[57] Abiodun O.I., Jantan A., Omolara A.E., Dada K.V., Mohamed N.A., Arshad H. (2018). State-of-the-art in artificial neural network applications: A survey. Heliyon.

[58] Ioffe S., Szegedy C. (2015). Batch normalization: Accelerating deep network training by reducing internal covariate shift. Proceedings of the 32nd International Conference on Machine Learning, ICML.

[59] Qi X., Chen G., Li Y., Cheng X., Li C. (2019). Applying Neural-Network-Based Machine Learning to Additive Manufacturing: Current Applications, Challenges, and Future Perspectives. Engineering.

[60] Shen X., Yao J., Wang Y., Yang J. (2004). Density prediction of selective laser sintering parts based on artificial neural network. Lect. Notes Comput. Sci..

[61] Li X., Dong J., Zhang Y. Modeling and applying of RBF neural network based on fuzzy clustering and pseudo-inverse method. Proceedings of the International Conference on Information Engineering and Computer Science.

[62] Munguía J., Ciurana J., Riba C. (2009). Neural-network-based model for build-time estimation in selective laser sintering. Proc. Inst. Mech. Eng. Part B J. Eng. Manuf..

[63] Wang R., Gutierrez-Farewik E.M. (2009). The effect of excessive subtalar inversion/eversion on the dynamic function of the soleus and gastrocnemius during the stance phase. Proceedings of the ASME Summer Bioengineering Conference 2009.

[64] Wang C.Y., Jiang N., Chen Z.L., Chen Y., Dong Q. (2015). Prediction of sintering strength for selective laser sintering of polystyrene using artificial neural network. J. Donghua Univ..

[65] Lee S.H., Park W.S., Cho H.S., Zhang W., Leu M.C. (2001). A neural network approach to the modelling and analysis of stereolithography processes. Proc. Inst. Mech. Eng. Part B J. Eng. Manuf..

[66] Caiazzo F., Caggiano A. (2018). Laser Direct Metal Deposition of 2024 Al Alloy: Trace Geometry Prediction via Machine Learning. Materials.

[67] Sood A.K., Equbal A., Toppo V., Ohdar R.K., Mahapatra S.S. (2012). An investigation on sliding wear of FDM built parts. CIRP J. Manuf. Sci. Technol..

[68] Vosniakos G.C., Maroulis T., Pantelis D. (2007). A method for optimizing process parameters in layer-based rapid prototyping. Proc. Inst. Mech. Eng. Part B J. Eng. Manuf..

[69] Equbal A., Sood A.K., Ohdar R.K., Mahapatra S.S. (2011). Prediction of dimensional accuracy in fused deposition modelling: A fuzzy logic approach. Int. J. Product. Qual. Manag..

[70] Sood A.K., Ohdar R.K., Mahapatra S.S. (2010). Parametric appraisal of fused deposition modelling process using the grey Taguchi method. Proc. Inst. Mech. Eng. Part B J. Eng. Manuf..

[71] Chen H., Zhao Y. Learning algorithm based modeling and process parameters recommendation system for binder jetting additive manufacturing process. Proceedings of the International Design Engineering Technical Conferences and Computers and Information in Engineering Conference.

[72] Everton S.K., Hirsch M., Stavroulakis P.I., Leach R.K., Clare A.T. (2016). Review of in-situ process monitoring and in-situ metrology for metal additive manufacturing. Mater. Des..

[73] Wasmer K., Le-Quang T., Meylan B., Shevchik S.A. (2019). In Situ Quality Monitoring in AM Using Acoustic Emission: A Reinforcement Learning Approach. J. Mater. Eng. Perform..

[74] Shevchik S.A., Kenel C., Leinenbach C., Wasmer K. (2018). Acoustic emission for in situ quality monitoring in additive manufacturing using spectral convolutional neural networks. Addit. Manuf..

[75] Zhang Y., Hong G.S., Ye D., Zhu K., Fuh J.Y.H. (2018). Extraction and evaluation of melt pool, plume and spatter information for powder-bed fusion AM process monitoring. Mater. Des..

[76] Scime L., Beuth J. (2018). A multi-scale convolutional neural network for autonomous anomaly detection and classification in a laser powder bed fusion additive manufacturing process. Addit. Manuf..

[77] Scime L., Beuth J. (2018). Anomaly detection and classification in a laser powder bed additive manufacturing process using a trained computer vision algorithm. Addit. Manuf..

[78] Scime L., Beuth J. (2019). Using machine learning to identify in-situ melt pool signatures indicative of flaw formation in a laser powder bed fusion additive manufacturing process. Addit. Manuf..

[79] Jafari-Marandi R., Khanzadeh M., Tian W., Smith B., Bian L. (2019). From in-situ monitoring toward high-throughput process control: Cost-driven decision-making framework for laser-based additive manufacturing. J. Manuf. Syst..

[80] Ye D., Hsi Fuh J.Y., Zhang Y., Hong G.S., Zhu K. (2018). In situ monitoring of selective laser melting using plume and spatter signatures by deep belief networks. ISA Trans..

[81] Bin Maidin S., Campbell I., Pei E. (2012). Development of a design feature database to support design for additive manufacturing. Assem. Autom..

[82] Yao X., Moon S.K., Bi G. (2017). A hybrid machine learning approach for additive manufacturing design feature recommendation. Rapid Prototyp. J..

[83] Shi Y., Zhang Y., Baek S., De Backer W., Harik R. (2018). Manufacturability analysis for additive manufacturing using a novel feature recognition technique. Comput. Aided Des. Appl..

[84] Zhang X., Le X., Panotopoulou A., Whiting E., Wang C.C.L. (2015). Perceptual models of preference in 3D printing direction. ACM Trans. Graph..

[85] Williams G., Meisel N.A., Simpson T.W., McComb C. (2019). Design repository effectiveness for 3D convolutional neural networks: Application to additive manufacturing. J. Mech. Des. Trans. ASME.

[86] Khadilkar A., Wang J., Rai R. (2019). Deep learning–based stress prediction for bottom-up SLA 3D printing process. Int. J. Adv. Manuf. Technol..

[87] Koeppe A., Hernandez Padilla C.A., Voshage M., Schleifenbaum J.H., Markert B. (2018). Efficient numerical modeling of 3D-printed lattice-cell structures using neural networks. Manuf. Lett..

[88] Chowdhury S., Anand S. (2016). Artificial Neural Network Based Geometric Compensation for Thermal Deformation in Additive Manufacturing Processes.

[89] Meng L., Zhao J., Lan X., Yang H., Wang Z. (2020). Multi-objective optimisation of bio-inspired lightweight sandwich structures based on selective laser melting. Virtual Phys. Prototyp..

[90] Gu G.X., Chen C.T., Richmond D.J., Buehler M.J. (2018). Bioinspired hierarchical composite design using machine learning: Simulation, additive manufacturing, and experiment. Mater. Horiz..

[91] Gu G.X., Chen C.T., Buehler M.J. (2018). De novo composite design based on machine learning algorithm. Extrem. Mech. Lett..

[92] Zohdi T.I. (2018). Dynamic thermomechanical modeling and simulation of the design of rapid free-form 3D printing processes with evolutionary machine learning. Comput. Methods Appl. Mech. Eng..

[93] Gan Z., Li H., Wolff S.J., Bennett J.L., Hyatt G., Wagner G.J., Cao J., Liu W.K. (2019). Data-Driven Microstructure and Microhardness Design in Additive Manufacturing Using a Self-Organizing Map. Engineering.

[94] Petrov R.A., Pernot J.-P., Giannini F., Falcidieno B., Véron P. (2016). Mapping Aesthetic Properties to 3D Free Form Shapes through the Use of a Machine Learning Based Framework.

[95] Sarlo R., Tarazaga P.A. (2016). A neural network approach to 3D printed surrogate systems. Topics in Modal Analysis & Testing.

[96] Wang R.J., Li J., Wang F., Li X., Wu Q. (2009). ANN model for the prediction of density in Selective Laser Sintering. Int. J. Manuf. Res..

[97] Pasquet I., Baco-Carles V., Chamelot P., Gibilaro M., Massot L., Tailhades P. (2020). A multimaterial based on metallic copper and spinel oxide made by powder bed laser fusion: A new nanostructured material for inert anode dedicated to aluminum electrolysis. J. Mater. Process. Technol..

[98] Yu W., Sing S.L., Chua C.K., Tian X. (2019). Influence of re-melting on surface roughness and porosity of AlSi10Mg parts fabricated by selective laser melting. J. Alloys Compd..

[99] Kuo C.N., Chua C.K., Peng P.C., Chen Y.W., Sing S.L., Huang S., Su Y.L. (2020). Microstructure evolution and mechanical property response via 3D printing parameter development of Al–Sc alloy. Virtual Phys. Prototyp..

[100] Ding D., Pan Z., Cuiuri D., Li H., Van Duin S., Larkin N. (2016). Bead modelling and implementation of adaptive MAT path in wire and arc additive manufacturing. Robot. Comput. Integr. Manuf..

[101] Ding D., Shen C., Pan Z., Cuiuri D., Li H., Larkin N., Van Duin S. (2016). Towards an automated robotic arc-welding-based additive manufacturing system from CAD to finished part. CAD Comput. Aided Des..

[102] Khaw J.F.C., Lim B.S., Lim L.E.N. (1995). Optimal design of neural networks using the Taguchi method. Neurocomputing.

[103] Xiong J., Zhang G., Hu J., Wu L. (2014). Bead geometry prediction for robotic GMAW-based rapid manufacturing through a neural network and a second-order regression analysis. J. Intell. Manuf..

[104] Mohamed O.A., Masood S.H., Bhowmik J.L. (2016). Investigation of dynamic elastic deformation of parts processed by fused deposition modeling additive manufacturing. Apem J..

[105] Bayraktar Ö., Uzun G., Çakiroğlu R., Guldas A. (2017). Experimental study on the 3D-printed plastic parts and predicting the mechanical properties using artificial neural networks. Polym. Adv. Technol..

[106] Asadi-Eydivand M., Solati-Hashjin M., Fathi A., Padashi M., Abu Osman N.A. (2016). Optimal design of a 3D-printed scaffold using intelligent evolutionary algorithms. Appl. Soft Comput. J..

[107] Zhu Y., Wu Z., Hartley W.D., Sietins J.M., Williams C.B., Yu H.Z. (2020). Unraveling pore evolution in post-processing of binder jetting materials: X-ray computed tomography, computer vision, and machine learning. Add. Manuf..

[108] Fang Z., Wang R., Wang M., Zhong S., Ding L., Chen S. (2020). Effect of reconsideration algorithm on the identification of 3D printing polymers on hyperspectral CT technology combined with artificial neural network. Materials.

[109] Boillat E., Kolossov S., Glardon R., Loher M., Saladin D., Levy G. (2004). Finite element and neural network models for process optimization in selective laser sintering. Proc. Inst. Mech. Eng. Part B J. Eng. Manf..

[110] Ahmadi A., Mirzaeifar R., Moghaddam N.S., Turabi A.S., Karaca H.E., Elahinia M. (2016). Effect of manufacturing parameters on mechanical properties of 316L stainless steel parts fabricated by selective laser melting: A computational framework. Mater. Des..

[111] Snell R., Tammas-Williams S., Chechik L., Lyle A., Hernández-Nava E., Boig C., Panoutsos G., Todd I. (2020). Methods for Rapid Pore Classification in Metal Additive Manufacturing. JOM.

[112] Cho H.S., Park W.S., Choi B.W., Leu M.C. (2000). Determining optimal parameters for stereolithography processes via genetic algorithm. J. Manuf. Syst..

[113] He H., Yang Y., Pan Y. (2019). Machine learning for continuous liquid interface production: Printing speed modelling. J. Manuf. Syst..

[114] Shamsaei N., Yadollahi A., Bian L., Thompson S.M. (2015). An overview of direct laser deposition for additive manufacturing; part II: Mechanical behavior, process parameter optimization and control. Addit. Manu..

[115] Saqib S., Urbanic R.J., Aggarwal K. Analysis of laser cladding bead morphology for developing additive manufacturing travel paths. Proceedings of the 47th CIRP Conference on Manufacturing Systems.

[116] Zhang W., Mehta A., Desai P.S., Fred Higgs C. Machine Learning Enabled Powder Spreading Process Map for Metal Additive Manufacturing (AM). Proceedings of the 28th Annual International Solid Freeform Fabrication Symposium.

[117] Tak J., Kantemur A., Sharma Y., Xin H. (2018). A 3-D-printed W-band slotted waveguide array antenna optimized using machine learning. IEEE Antennas Wirel. Propag. Lett..

[118] Wang T., Kwok T.H., Zhou C., Vader S. (2018). In-situ droplet inspection and closed-loop control system using machine learning for liquid metal jet printing. J. Manuf. Syst..

[119] Kwon O., Kim H.G., Ham M.J., Kim W., Kim G.H., Cho J.H., Kim N., Kim K. (2020). A deep neural network for classification of melt-pool images in metal additive manufacturing. J. Intell. Manuf..

[120] Pant M., Singari R.M., Arora P.K., Moona G., Kumar H. (2020). Wear assessment of 3-D printed parts of PLA (polylactic acid) using Taguchi design and Artificial Neural Network (ANN) technique. Mater. Res. Express.

[121] Ahmadi Dastjerdi A., Movahhedy M.R., Akbari J. (2017). Optimization of process parameters for reducing warpage in selected laser sintering of polymer parts. Addit. Manuf..

[122] Fountas N.A., Kostazos P., Pavlidis H., Antoniou V., Manolakos D.E., Vaxevanidis N.M. (2020). Experimental investigation and statistical modelling for assessing the tensile properties of FDM fabricated parts. Procedia Structural Integrity.

[123] Fountas N.A., Kechagias J.D., Manolakos D.E., Vaxevanidis N.M. (2020). Single and multi-objective optimization of FDM-based additive manufacturing using metaheuristic algorithms. Procedia Manuf..

[124] Géron A. (2019). Hands-On Machine Learning with Scikit-Learn, Keras, and TensorFlow: Concepts, Tools, and Techniques to Build Intelligent Systems.

[125] Kingma D.P., Welling M. Auto-encoding variational bayes. Proceedings of the 2nd International Conference on Learning Representations, ICLR 2014—Conference Track Proceedings.

[126] Goodfellow I.J., Pouget-Abadie J., Mirza M., Xu B., Warde-Farley D., Ozair S., Courville A., Bengio Y. (2014). Generative Adversarial Nets. arXiv.

[127] Makhzani A., Shlens J., Jaitly N., Goodfellow I., Frey B. (2015). Adversarial Autoencoders. arXiv.

[128] Yusuf S.M., Gao N. (2017). Influence of energy density on metallurgy and properties in metal additive manufacturing. Mater. Sci. Technol..

[129] Srivastava N., Hinton G., Krizhevsky A., Sutskever I., Salakhutdinov R. (2014). Dropout: A simple way to prevent neural networks from overfitting. J. Mach. Learn. Res..

[130] Ng A.Y. (2004). Feature selection, L1 vs. L2 regularization, and rotational invariance. Proceedings of the Twenty-First International Conference on Machine Learning, ICML 2004.

[131] Online Materials Information Resource—MatWeb. http://www.matweb.com/.

